# Responsive Dashboard as a Component of Learning Analytics System for Evaluation in Emergency Remote Teaching Situations

**DOI:** 10.3390/s21237998

**Published:** 2021-11-30

**Authors:** Emilia Corina Corbu, Eduard Edelhauser

**Affiliations:** 1 Department of Mathematics and Informatics, University of Petrosani, 332003 Petrosani, Romania; corinacorbu@upet.ro; 2 Department of Management and Industrial Engineering, University of Petrosani, 332003 Petrosani, Romania

**Keywords:** online education, learning analytics, emergency remote teaching, responsive dashboard, eLearning and digital transformation of education, IT for education

## Abstract

The pandemic crisis has forced the development of teaching and evaluation activities exclusively online. In this context, the emergency remote teaching (ERT) process, which raised a multitude of problems for institutions, teachers, and students, led the authors to consider it important to design a model for evaluating teaching and evaluation processes. The study objective presented in this paper was to develop a model for the evaluation system called the learning analytics and evaluation model (LAEM). We also validated a software instrument we designed called the EvalMathI system, which is to be used in the evaluation system and was developed and tested during the pandemic. The optimization of the evaluation process was accomplished by including and integrating the dashboard model in a responsive panel. With the dashboard from EvalMathI, six online courses were monitored in the 2019/2020 and 2020/2021 academic years, and for each of the six monitored courses, the evaluation of the curricula was performed through the analyzed parameters by highlighting the percentage achieved by each course on various components, such as content, adaptability, skills, and involvement. In addition, after collecting the data through interview guides, the authors were able to determine the extent to which online education during the COVID 19 pandemic has influenced the educational process. Through the developed model, the authors also found software tools to solve some of the problems raised by teaching and evaluation in the ERT environment.

## 1. Introduction

During the pandemic crisis, governments from various countries decided to force the closure of educational institutions and universities by implementing new learning models that could help the education sector to continue its work exclusively online. Several types of teaching were adopted, including mobile learning and blended learning. According to UNESCO, only 20% of countries globally were equipped with online teaching devices and programs before the pandemic [1].

In the academic year of 2020/2021, a pandemic year, the world’s universities were forced, in most cases, to cancel face-to-face courses and to use online education. The responses of European countries differed, and online education took place to varying degrees—25%, 50%, 75%, or 100%, depending on local conditions. Alternating distance learning with present or face-to-face learning (F2F) has been the subject of several studies [2,3].

The drastic changes imposed by the pandemic have had a multitude of effects on learning environments, including open ones. Learning environments have undergone a changing trend from learning management systems to personal learning environments [4], and in terms of learning infrastructures, they have provided learning services from open educational resources to a classroom framework [5]. In the UK, according to the YouGov study in February 2021, it was shown that the adaptation of educational tools was achieved by using video conferencing applications such as Zoom, Google Meet, and Microsoft Teams, and for communication with students, Microsoft Teams and Google Classroom were used as collaborative platforms.

Teaching exclusively online has raised a multitude of issues, namely video transmission problems, slow access to platforms, untimely responses to questions in class, students’ hopes to get corresponding guidance after class, and teachers’ feedback communicated in a timely manner so as to improve the students’ learning. Considering the resources used in multiple studies, proposals were made to use online resources for as many courses as possible, as well as to add as many activities as possible to online platforms to satisfy as many students as possible [6].

The learning analytics system through embedded dashboards was used by the authors to monitor the activities that generate the learning process. It was also used in the monitored activities, by analyzing algorithms using educational data mining techniques [7] and viewing information, to monitor the degrees of involvement and reflection.

The study was based on the proposed model and the designed and implemented application, and it was conducted in regard to the educational process in crisis situations or ERT situations, that is, online teaching and evaluation, representing an element of novelty in the field of online educational resources. Training carried out under the pressure of time with minimal resources is called emergency remote teaching (ERT). Through the application developed, the authors tried to solve some of the problems that this type of teaching raises. There is still the fear that by the end of 2021, or after a certain period of time, the pandemic experience will be repeated, which is why the authors focused on developing a package of software tools. Regarding the evaluation, in the second semester of the 2019/2020 academic year, which was carried out exclusively online, the authors thought about the need to develop tools that help teachers in teaching, both in the process of evaluating courses and evaluating the students. For this purpose, a model was designed for the evaluation, a system called the EvalMathI system. The EvalMathI system is software that supports teachers in their teaching activities, and it can be used for two evaluation processes, namely course evaluation and student evaluation. From the designed software, the dashboard tools necessary for monitoring and evaluating several disciplines were implemented and tested. In addition, an optimization module was added by introducing and integrating the dashboard into a responsive panel to facilitate and streamline the evaluation process.

The results of the study are based on the answers of the students involved in an investigation conducted by both authors. The sample was composed of 157 students in the 2019/2020 academic year and 143 students in the 2020/2021 academic year. Questionnaires were distributed, and 190 and 339 answered the survey each year, respectively. The students who responded can be considered representative from the perspective of the tested model and application.

The process of monitoring and the evaluation of the six courses through the EvalMathI dashboard were also attended by nine teachers from the three faculties of the University of Petrosani, the university where the study took place, namely four from the Faculty of Mining, three from the Faculty of Sciences, and two from the Faculty of Mechanical and Electrical Engineering. For each of the six monitored courses, tools were developed for the evaluation of the discipline through the parameters analyzed, highlighting the percentage achieved by each course on various components.

By elaborating the learning analytics and evaluation model (LAEM) and the proposed application—the EvalMathI system—the authors managed to coordinate the monitoring and evaluation activity of the six optional courses carried out with 300 students. Throughout this study, the authors aimed to answer the following questions regarding the central objective by evaluating the utility of the proposed learning analytics and evaluation model (LAEM) as well as the efficiency of the EvalMathI system software application:

Q1—How did EvalMathI affect the evaluation process of the courses in an ERT situation?
Q2—What are the best EvalMathI dashboard tools regarding content evaluation?
Q3—How is the content of each course assessed through EvalMathI in terms of relevance and applied scientific content, coherence, and consistency?
Q4—What skills were obtained by the students in completing the six courses?

## 2. Related Work

### 2.1. Emergency Remote Teaching (ERT) and Learning System Process

Lately, the teaching–learning process has been the subject of many studies; previous research has referred to different processes as blended learning, face-to-face learning, or eLearning. Because of the COVID-19 threat, universities and colleges have had to decide how they can continue teaching under conditions of acute uncertainty. Many institutions have opted to suspend face-to-face classes, including the operation of laboratories, opting for online courses.

In a normal situation, the planning, preparation, and elaboration of a completely online university course requires an elaboration time between six and nine months, time that the teachers did not have during the pandemic [8]. It was also impossible for every university professor to suddenly become an expert in online teaching and learning. The process of preparing and implementing online resources was prolonged over time, increasing stress and pressure, and it was sincerely acknowledged that there were many online teaching experiences in which instructors failed to fully prepare materials, leading to a large probability of suboptimal implementation [9].

In this time of crisis, teachers and students have made the best use of the available resources, but it must be acknowledged that there is a big difference between vocational training on online platforms and education carried out under the pressure of time with minimum resources—emergency remote teaching (ERT) [8].

In contrast to activities that are planned and designed from the beginning to be online, emergency remote teaching is a temporary form of education that is used to carry out the training process in an alternative way in crisis circumstances. This mode of training involves the use of entirely remote teaching solutions. The main objective is not to create a robust educational ecosystem but rather to ensure temporary access to training in a rapid manner in an emergency or in a period of emergency crisis. This is the main difference between ERT and classic online learning [8].

During the pandemic, some educational institutions supplemented the hardware support made available to teachers for conducting online courses. The technical staff of universities were also involved to facilitate the educational process. Carrying out all activities exclusively in an online environment meant flexibility in the teaching and learning processes [10,11], but the problems raised by teaching exclusively online were multiple because many of the systems that provided the resources were overworked and even exceeded capacity [9].

In ERT, speed and “just get it online” are detrimental to the quality of the course, so the authors of [9,10,11] believe that courses created in an ERT situation should be a temporary solution to an immediate problem. Further, the principles of the universal design for learning (UDL) should focus on creating flexible, inclusive, student-centered learning environments that ensure that all students have access to courses and activities [12].

### 2.2. Learning Analytics Dashboards and Learning Analytic Evaluation Models

Siemens (2010) defines learning analytics as “the use of intelligent data, learner-produced data, and analysis models to discover information and social connections, and to predict and advise on learning”. In recent years, a series of tools have been developed to monitor and/or to evaluate learning activities and then to visualize them in learning dashboards. Learning dashboards, depending on how the teaching process is carried out, are divided into three categories—dashboards based on face-to-face courses, dashboards for face-to-face work groups, and dashboards that work in online learning or blending learning [13,14].

Dashboards that collect data from traditional face-to-face courses have been integrated into multiple applications, such as Backstage [15], which is a dashboard that displays Twitter activity during the course activities [16], Classroom Salon [17] which allows teachers to create, manage, and analyze social networks to view the contribution of each member, and in which the dashboard allows for viewing the contribution of each member to view the correct answers received, and finally, Slice 2.0. [18], which is a system that interconnects the teacher’s slides with the students‘ devices, and the teacher can view the students’ annotations.

Several dashboards for working in face-to-face groups and a classroom orchestration design [19] have been developed, including TinkerBoard [20], Collaid [21], and Class-on [22,23,24], and some of them were developed by taking data from OpenSocial [25].

A learning analytic evaluation model usually contains the following groups of parameters: the relevant student actions, captured data on relevant actions, awareness, reflection, sense-making, and impact, and effectiveness, efficiency, and usefulness.

1. Relevant student actions. In face-to-face teaching, time and date, location, who and what kind of device they use, and even the background sounds were analyzed, and each item has a greater or smaller significance. Students find that social interaction is somewhat useful, especially for blended or face-to-face courses [26]. Teachers consider the visualization of social interaction more important because it is useful in identifying students who do not collaborate with others or those who collaborate excessively. Teachers consider that the effort of students is very important. However, the usefulness is seen differently by teachers and students. Students often believe that the data collected does not reflect the effort made, while teachers perceive the data to be useful for an in-depth view and possibly for the identification of students potentially at risk. All the data mentioned above refer to quantitative data, which is why it was investigated whether it was possible to increase the qualitative data, such as the number of re-tweets or comments on a blog post, and whether that can indicate the relevance of such communication. This idea was already explored by the authors of the Backstage dashboard [16].

2. Capture data on relevant actions. Many learning analytic dashboards take data from virtual sensors through certain tools and resources, such as laptop or desktop user interactions and social media, through hashtags or blog comments. One of the main problems tracking learning activities, an aspect called automated tracking. Previous studies have shown that students rate the usefulness of dashboards as low when some of the activities covered take place outside of the learning environment [27]. Regarding personal learning environments, which include a wide variety of tools and services that aggregate data from various sources—Twitter, blog posts, comments, software environments, and so on—the feedback is positive. The use of sensors such as cameras or microphones to capture student data for monitoring and counseling activities is the subject of computer-supported collaborative learning (CSCL) research. They are present to a small extent in learning dashboard applications because they provide real-time feedback to students or teachers [28].

3. Awareness, reflection, sense-making, and impact. Bakker et al. [29] presented research on the use of sensors to capture physiological responses and to both estimate stress levels and provide feedback to employees on their current work schedule. Such research on the level of awareness and reflection in learning environments and the impact of such awareness have been presented at the Learning Analytics and Knowledge (LAK) conference [30,31].

4. Effectiveness, efficiency and, utility. Efficacy has been measured in terms of better engagement [24,32], higher grades [26,33,34], post-test results [34], lower retention rates [26], and improved self-assessment [35]. The results of a long-term experiment regarding course signal [26] indicated that there is an impact on retention rates and grades. There was also a significant difference in improving self-assessment in an evaluation of the CALMS system [36]. An evaluation of TADV [20] indicated that the overall satisfaction with the course for students using the dashboard was higher, satisfaction that was measured in terms of self-esteem and the recommendation of the course to other students. In other experiments, student involvement has been measured to obtain information on the potential impact. The results of Morris et al. [24] indicated that there was no increase in involvement when learners used the scoreboard. The results of iTree [35] indicated that the dashboard does not encourage learners to post messages on a forum, but there is an increase in the reading of posts. Efficiency was measured in a Class-on assessment experiment [23], which assessed whether using a dashboard during class sessions helped to distribute a teacher’s time more accurately. Usage and utility evaluations were performed—either by teachers or students, or both. The perceived usefulness of the Student Inspector [37] and LOCO-Analyst [21] dashboards was, for example, evaluated by teachers and was high for both categories of dashboard users. The results of the evaluations for SAM and StepUp indicated that the perceived utility is often greater for teachers than for students [38]. The results of the LOCO-Analyst assessment [21] also indicated that the perceived utility was significantly higher in a case study in which several data points were used to provide a perspective on the learning activity.

### 2.3. Dashboards for Online Learning

For retrieving data from an online environment, dashboards for online learning have been created for online learning and blended learning. Course signals [26] predict and visualize learning outcomes based on three data sources—grades in the course so far, time spent on a task, and past performance.

The dashboard developed by Carnegie Mellon University [39] is highly detailed, and concepts and how they are carried out on different course activities may need additional attention from the student. Displaying various parameters differentiates dashboards for online learning. A student activity meter (SAM) [38] displays the progress of a particular course and illustrates the time spent by students in different study environments. LOCO-Analyst [40], the Moodle dashboard [41], and GLASS [42] are tools that visualize student feedback and different levels of performance in different ways. Student Inspector [43] visualizes the use of data in the Active Math environment. Tell Me More [44] provides visualizations of exercise results. The CALM system [45] visualizes comparative levels of knowledge through self-assessments [33].

There are models with components dedicated especially to students, such as Teacher Advisor [46], which is based on manual interventions to automatically generate tips for the student, or StepUp [47], which is a model designed for mobile devices for students who apply learning analytics techniques for awareness and self-reflection.

In their latest work, Vieira et al. [48] analyzed visual learning analytics and concluded that there are few studies that have simultaneously deepened complex visualizations and educational theories, a statement also supported by Jivet et al. [49], who analyzed learning dashboards from the students’ point of view.

From the category of learning dashboards used on cloud platforms [50], Amazon Web Services offers two solutions for monitoring—AWS CloudTrail resources [51], a managed service to track user activity and API usage, and Amazon CloudWatch, a monitoring service of cloud resources and applications. There are several workarounds on the market that offer more powerful dashboards for cloud monitoring, including Opsview Monitor [52], Spectrum [53], SignalFx [54], and AWS Cloud Monitoring [55]. However, these are all expensive enterprise solutions that are difficult to use in academic or education fields in countries like Romania.

Lonn, in one of the most recent studies focused on learning analytics tools that use dashboards that measure performance, stated that they may decrease learner mastery orientation and the students’ exposure to graphics of their academic performance may negatively affect the students’ interpretations of their own data as well as their subsequent academic success [56]. Thus, Sedrakyan et al. focused on feedback and its speed in certain activities during laboratory hours to improve the feedback given to students, and they did not focus on academic performance [57]. Irons et al. [58] stated that there is a direct proportionality between student feedback and impact, so the faster the feedback, the more substantial the impact in learning; thus, the ability to give timely feedback is very important.

From the analysis, it can be stated that it is a challenge to retrieve data on the evaluation process from emergency remote teaching, so one of the contributions of this study is that it proposes a tool that is actually a system that comes both in support of teachers as well as students working in an ERT situation. This tool was partially validated in the period of 2020/2021 at a university in Romania.

## 3. Materials and Methods

From the analysis performed and from the evaluation of the difficulties encountered in the teaching and evaluation process in ERT, the authors proposed a method of collecting data on the results of different teaching and evaluation activities, as well as their processing and subsequent visualization in a certain form of the activities monitored, minimizing the time required for corrections.

The proposed learning analytics and evaluation model (LAEM) was developed by the authors and is intended to be a support for teachers conducting ERT by collecting, integrating, and analyzing data from various sources through a semi-automated process. Subsequently, the designed software tool, the EvalMathI system is intended to be a support for professors at the University of Petrosani in their attempt to automate and streamline certain components of the evaluation process. EvalMathI proposes a solution for teachers and students to access information about courses by completing questionnaires regarding the evaluation process. EvalMathI was also tested according to the evaluation process, and the results indicate that it is a responsive dashboard.

In order to establish the component elements of the input data for the learning analytics block, data collected from the learning activities and data that evaluate the activities carried out on various platforms were analyzed on the basis of the project POCU 12596, implemented by the authors as project managers at the University of Petrosani, and financed by the European Commission through the Romanian Operational Program Human Capital (OPHC) [59]. The virtual class platform for the courses was analyzed, monitored, and evaluated [60,61].

### 3.1. The Learning Analytics and Evaluation Model (LAEM) Design, Based on Virtual Sensors

**Figure 1:**
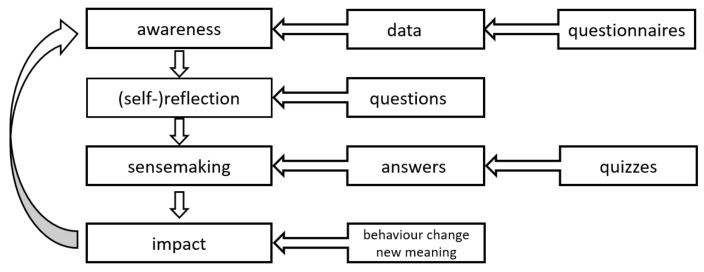
The learning analytics process model adaptation of [62].

In developing the model for the proposed assessment system, the learning analytics process model (LAPM) proposed by Verbert, K. [62] was adapted by the authors, and the modified model is detailed in Figure 1, model in which the project’s groups of parameters are represented.

This model was customized by the authors for the conditions in which the teaching–learning process took place by ERT at the University of Petrosani in the two pandemic years. In developing the LAEM model, the authors analyzed the following groups of parameters: the relevant student actions, captured data on relevant actions, awareness, reflection, sense-making, and impact, and effectiveness, efficiency, and usefulness, all of which were captured by virtual sensors. Then, the groups of parameters analyzed in the LAPM were customized for LAEM as well as the EvalMathI system.

In developing the LAEM model for the design of the EvalMathI system, the time and date, the location, who and what kind of device they use, the background noise, the use of resources, and the results of tests and exercises were taken into account. These results are the main data taken from the EvalMathI system dashboard to be a support in the evaluation process performed by teachers. The LAEM model that generated the EvalMathI system dashboard retrieves data from virtual sensors through laptop or desktop interactions. In applying LAEM and EvalMathI system customization for this parameter, we tried to assess awareness by measuring the feedback received in the virtual classroom, but this developed system will be the subject of further research.

This study answered questions related to the evaluation of courses conducted in an ERT situation through our own design, EvalMathI, software that displays the status of indicators monitored for the evaluated courses in a responsive dashboard.

The tool does not evaluate the students’ activity at this moment, but the authors intend to further develop that part as well. Thus, regarding the analysis performed on existing solutions, the authors considered the possibilities of collecting data from the virtual classes and implementing a virtual sensor for them.

Customizing the LAEM and EvalMathI system for this parameter measured the effectiveness from the perspective of the students because they obtained better results in the tests measured in the second series of courses, demonstrating the efficiency and usefulness of EvalMathI.

**Figure 2:**
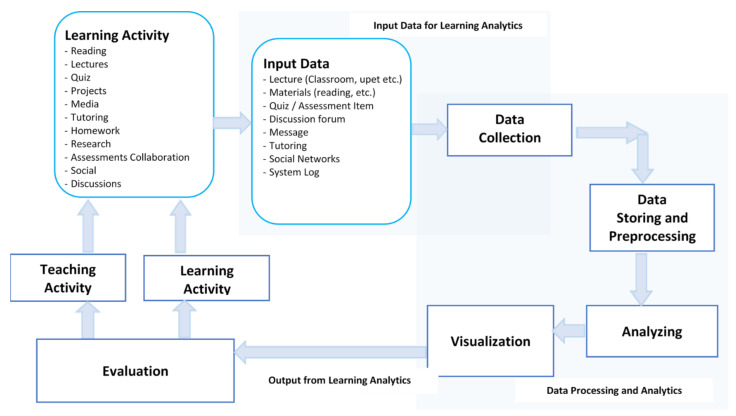
The learning analytics and evaluation model proposed by the authors for designing the EvalMathI system in ERT cases, based on virtual sensors.

The conceptual scheme of the learning analytics and evaluation model presented in Figure 2 applied in ERT was elaborated by the authors. In this model, the learning activities included reading, lectures, quizzes, projects, media, tutoring, homework, research, assessments collaboration, social media, and discussions, resulting as input data for learning analytics.

Following the research carried out on various learning models, starting with analysis, design, development, implementation, and evaluation (ADDIE) [43], the authors established the main stages of the process. In the next stage, the authors analyzed the proposed CIPP evaluation model [12] and the proposed adaptive learning model [37]. The authors proposed the model from Figure 2 as the best for exclusively online courses in ERT.

The learning analytics and evaluation model proposed for the EvalMathI system, and schematically presented in Figure 2, shows the way in which data taken from learning activities constitute input data in the data collection. The main learning activities that the projected model considers are reading, lectures, quizzes, projects, media, tutoring, homework, research, assessments collaboration, social media, and discussions. From these activities, the input data block consists of lectures, materials, quiz/assessment items, discussion forms, messages, tutoring, social networks, and data from the system log. The data processing and analytics block incorporates the data collection and storing, and the preprocessing, analyzing, and visualization. The output of this block constitutes the input data for the evaluation block. The evaluation block analyzes the data from the learning analytics and sends an indicator of the situation at a certain course to the teaching activity as well as one indicator to the learning activity. Each of the two activities should be rethought or corrected in order to increase the indicator’s value.

### 3.2. Design and Development of the EvalMathI System Software

The proposed LAEM model was validated by the EvalMathI system, a component of which helps teachers in evaluation processes, which is why the LAEM model was used and the application was designed and developed. The EvalMathI system is a web-based application that can be used by students and teachers. The application also allows for testing as well as disciplinary evaluation and monitoring.

For the database, in the 2020/2021 academic year, the authors used the MySQL database for teachers, students, and the courses created; it will be extended later to the administrative staff. For the development of the EvalMathI system, technologies were used for indexing, searching, and analyzing the data available under the Apache server, Apache 3.2.4 Open Source License [63].

**Figure 3:**
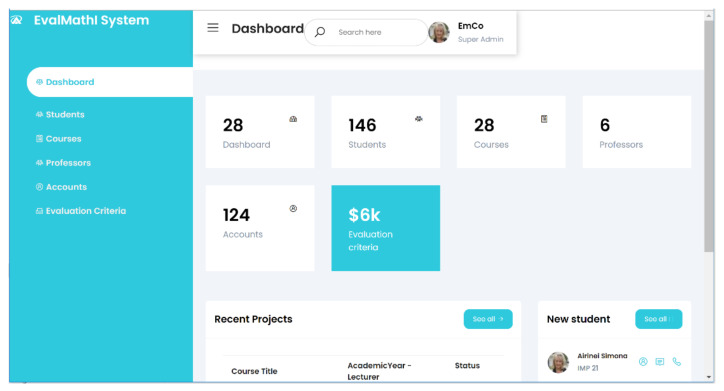
EvalMathI system admin panel and main page.

The EvalMathI system admin panel and the main responsive are presented in Figure 3, representing two solutions. Each of the two solutions performs different sections in the evaluation process. EvalMathI was developed in PHP and collects data from teachers and students using pages created for each type of user. The evaluations made by the experts are collected using Elasticsearch and are then processed with Kibana.

From a structural point of view, the EvalMathI system has access to the MySQL databases of students, teachers, and courses. It provides aggregated information regarding the use of resources at a given time in a given framework by a particular user, detailed information about a specific activity carried out by a specific student in a specific setting, and the percentage of progress made for a certain course in a certain moment of time, on certain parameters studied, evaluated by a group of experts with well-defined roles in the activity.

Users with a teacher profile can view each student’s progress in a particular course. The scores obtained for tests taken and the degree of completion of each session can be tracked. The benefits of the dashboard for this profile include the monitoring of several students at a time, providing automatic feedback from students as well as obtaining useful parameters for evaluation.

Users with a student profile can view the evaluation of their progress for each test taken, the tests they still have to complete if the instructor agrees, and a comparison of their progress with the rest of the class. The benefits of the dashboard to this profile include self-regulated learning, planned learning, thinking and evaluating tasks and contexts [64], motivation [65], and an overview of the class.

The EvalMathI system offers users with the system administrator profile the ability to view detailed or aggregated resource consumption, to view history, use resources, and monitor current resource consumption. Elasticsearch, a popular and powerful distributed search and analytics engine based on Apache Lucene and designed for horizontal scalability, reliability, and easy management, was designed to further connect the system to other databases. It combines the speed of searching with the power of analytics via a sophisticated, developer-friendly query language, covering structured, unstructured, and time series data [66]. For the visualization and presentation, Chart.js was used, a visualization platform that allows for the interaction between the graphs, and the Kibana tool [67] was used for the subsequent developments of the application that will include histograms and geo maps.

### 3.3. Data Collection through the Activity Analysis Sheet (AAS) and its Validation

Nine teachers were used as evaluators from the three faculties of the University of Petrosani. They participated in the monitoring and evaluation processes of the six courses using the EvalMathI dashboard. Four were from the Faculty of Mines, three from the Faculty of Sciences, and two from the Faculty of Mechanical and Electrical Engineering. Six optional courses were monitored, namely Sustainable Development, Creativity and Innovation, Environmental Management, Renewable Energy, Cyber Security, and Web Programming. These optional courses were attended by students and master students, with 157 students in the 2019/2020 academic year and 143 students in the 2020/2021 academic year.

#### 3.3.1. Instruments and Investigation Tools

An online questionnaire was the investigation tool selected for this study, as an online survey was the most significant and reliable method for conducting this study. The collected data were processed by elaborating the structure of the data matrix and encoding the answers of the questionnaire applied. The results of the questionnaires were processed using the IBM SPSS Statistics application, version 23, with which the variables were also verified.

The study was based on three online questionnaires. Two of them were given to the students, the “Questionnaire for evaluating the teaching activity”, composed of 17 questions, and “Course observation”, composed of 31 questions. One was distributed to the teachers—“Activity product analysis”, composed of 31 questions.

The two surveys completed by the students took place over two stages. The first one was completed by 157 students in May 2020, and the second one was completed by 143 students in March–May 2021. The 300 students involved in the POCU 122596 project were monitored during six online courses carried out within the project. The students chose one or more of these six courses, and the total number of students involved in our survey was 529.

The survey completed by the teachers who presented the six courses took place during the same period and a group of nine experts, teachers—also called evaluators—participated during the same POCU 122596 project. Each of the nine experts evaluated the curricula, the teaching, and the evaluation system of the three courses in six different stages of the project, for a total of 162 responses.

#### 3.3.2. Questionnaire Validation

By using the three proposed questionnaires, the authors’ intention was to evaluate the curricula of six courses used in an online teaching and evaluation system through seven parameters—Content Design Intra- and Interdisciplinary Relationship (CDDR), Content Design Intra and Intercurricular Relationship (CDCR), Relevance for Life and Applied Scientific Content (RLASC), Degree of Structuring (DS), Degree of Systematization (DSY), Coherence (CH), and Consistency (CS). The percentages achieved by each course were determined regarding components such as content, adaptability, skills, or involvement.

The construct validity of the questionnaire was tested using the Pearson correlation matrix of major variables related to the online level. In addition, the fidelity and internal consistency of the questionnaire were tested using the Cronbach alpha for the multiple Likert questions.

After receiving the data from the respondents, they were processed accordingly using inferential statistics such as the Cronbach alpha coefficient to assess reliability. These statistical analysis tools were used to process the questionnaires given to the target group. To transform the information gathered with the questionnaires, the authors used the variables in SPSS—nominal, ordinal variables, which are qualitative variables, and the range and ratio variables, which are quantitative. To assess the reliability, the Cronbach coefficient was used; as indicated by Sekaran, a Cronbach alpha coefficient of 0.70 or higher is considered reliable and acceptable.

The authors assessed the reliability using the Cronbach alpha coefficient for the main variables. A value of 0.829 indicates a high level of internal consistency in this study with this specific sample. The results for the seven parameters that evaluated the curricula and the correlation matrix show the strength of the association between the variables, as demonstrated in Table 1. The Cronbach alpha coefficients for all variables were well above the threshold of 0.70, and it can be deduced that the results meet the reliability hypothesis and that the reflective constructs have sufficient reliability.

#### 3.3.3. Population and Sample—Respondents

This study was based on the responses of the students of two faculties of the University of Petrosani, the Science and the Mining Faculties. The total number of students of these two faculties was 2153. It is important to mention that the University of Petroșani has only three faculties; the authors selected the Science and the Mining Faculties because their teaching activities involve students from only these two faculties, so the students could be easily contacted, and as a result, their responses were representative. The 529 answers represent 25% of the total number of students, as can be seen in Table 2. The students of these two faculties who answered the questionnaire are students in the bachelor’s or master’s degree programs.

The study was based on the University of Petrosani, a small university that can be considered representative, not for the whole Romanian education system, but for the small Romanian universities. This assumption can be based on the fact that small universities are similar in the field of online education because, unfortunately, they started the massive implementation and use of eLearning only after 15 March 2020. The Romanian National Council for the Financing of Higher Education statistics provide the total number of Romanian students for the 2019/2020 academic year—459,899. The same statistics show that out of the total 49 Romanian State Universities, 32 are small universities with around 5000 students, representing 30% of Romanian students. On the other hand, these small universities are very important and representative because most of them are comprehensive universities with the most fundamental areas of study, such as engineering, social sciences, and humanities. The University of Petrosani is a very small university, with 3565 students and engineering and social science areas of study.

In the field of eLearning, all these small universities are similar; they did not previously have distance learning, so they had to adjust very quickly to the new conditions of education generated by the COVID-19 Pandemic. Additionally, these small universities could not afford to buy an eLearning platform produced by one of the major world players in this field. These small universities have adopted low-priced eLearning platforms, such as LMS, developed by Moodle, or free collaborative educational platforms.

This study was also based on the responses of the evaluators from the same university, teachers that evaluated other teachers during the development of the POCU 122596 project in the 2019/2020 and 2020/2021 academic years. Each of the nine experts evaluated the curricula, the teaching, and the evaluation system through seven main parameters—CDDR, CDCR, RLASC, DS, DSY, CH, and CS—for three courses at six different stages of the project, for a total of 162 results. This number of evaluation results could be approximated as 10% of the total number of courses at the University of Petrosani that are evaluated by one evaluator per course at one time.

#### 3.3.4. Questionnaire Description

To monitor the activity, we used a questionnaire called the “Activity Products Analysis Sheet” (APAS). The APAS was carried out in APAS version S1 and APAS version S2 for each series of students who participated in the courses. For the monitoring of the courses, the Analysis Sheet had the following components: (1) Analysis of the course content; (2) Adaptation to the requirements of the project; (3) Competences brought to the students; (4) Determination of the students’ activity; (5) Other items.

1. Regarding the content analysis, the analyzed parameters were whether the content design supports the intra- and interdisciplinary relationship, whether the content design supports the intra- and intercurricular relationships, whether the course design supports the lifelong relevance of the content, the applied scientific content, the degree of structuring, the degree of systematization, course coherence, and course consistency.

2. Regarding the adaptation to the project requirements, we analyzed the following parameters: to what extent it offers intellectual activity skills; to what extent it offers skills of applicative activity; it is correlated with the project objectives.

3. Regarding the competences taught to the students, the following parameters were monitored: to what extent the courses offer professional, social, or other competences.

4. In order to determine the students’ activity, the following parameters were monitored: the attitude towards learning; attendance at the course; the attitude and responsibility of the students towards solving the work tasks; collaboration in the learning process; the degree of use of knowledge, skills, and attitudes in new learning contexts; the progress made by the students during the course.

These parameters were not included in this course analysis and evaluation study because they were input data for the student evaluation. These parameters influenced the learning activities (LA) in the LAEM model and not the teaching activity. These parameters are described as the parameters of the evaluation block of the LAEM.

5. Other elements taken into account were the educational environment, the place where the course took place and the platform used; funding, material resources, and curricular auxiliaries used; presentation of materials, student work, the general atmosphere during the course, and other observations

In the questionnaires used at the first stage of the study, there were some questions that refer to these parameters, but they were not used in the present study. During the first stage of the study, the 2019/2020 academic year, the authors recorded data for these parameters, but the authors did not interpret them, as they were not considered representative.

## 4. Case Study Results: Dashboard for Courses Evaluation

### 4.1. EvalMathI System Dashboard for Courses Evaluation—Beta Version

The dashboard designed by the authors for the course evaluation was developed in the first stage in a beta version in the 2019/2020 academic year and tested in the first semester of the 2020/2021 academic year when the University of Petrosani transitioned all teaching activities to online in an emergency remote teaching (ERT) situation. In ERT, the courses were held in a video conference system, and the main platforms used were Zoom, Google Meet, and Microsoft Teams. The activities carried out during laboratory classes were developed in virtual classes created for each course. In the virtual classes, the activities carried out by the groups of students for each discipline were monitored. The student assessments were also conducted online by developing tests and quizzes in Microsoft Forms. The exams at the end of the first semester were taken by participating in a video conferencing system and completing quizzes.

**Figure 4:**
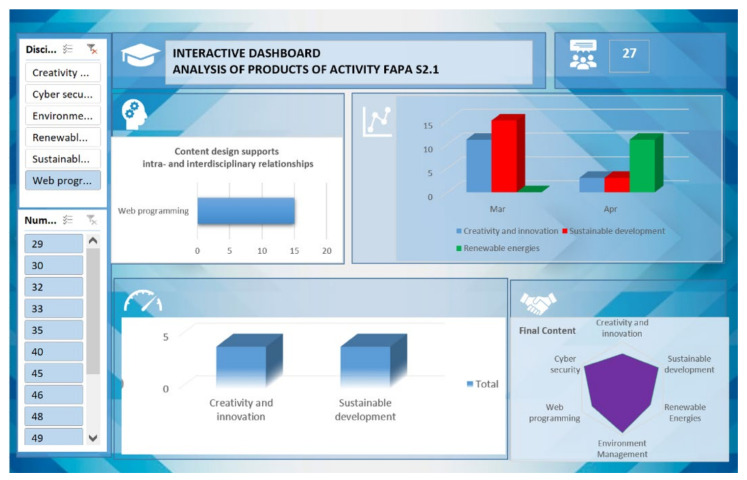
EvalMathI dashboard for courses panel—beta version.

The beta version of EvalMathI presented in Figure 4 was designed to evaluate six courses in the 2019/2020 academic year and contains filters that are suitable for different categories or fields. It can also offer information for a selected course.

### 4.2. EvalMathI System for Course Evaluation

The answers to the four questions, Q1, Q2, Q3, and Q4, which support the central objective of the study—the design and testing of an evaluation model under ERT condition—are presented as follows:

Q1. How did EvalMathI affect the evaluation process of the courses in an ERT situation?

For the interactive communication between teachers and students, the authors designed a window in the application with which users can interact to share information and communicate. Regarding the optimization use mode, an attempt was made to improve the stability, security, and compatibility of the platforms. Because the method of archiving information was scattered among a number of applications, and their unification required consistent effort, the authors thought it would be useful for the application to gather the information needed for an evaluation in a single window, and based on this information, the evaluation can be carried out. The optimization of the usage method was solved by inserting and integrating the dashboard in a responsive panel in order to facilitate and make the evaluation process more efficient.

#### 4.2.1. EvalMathI System Responsive Application Programing Interface (API) Optimization

**Figure 5:**
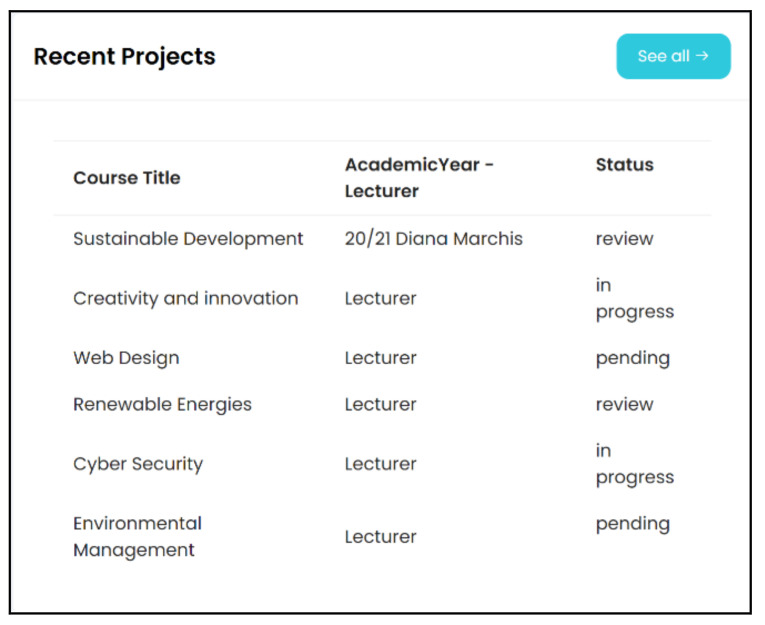
Responsive API EvalMathI—courses panel.

The responsive dashboard of the EvalMathI presented in Figure 5 was developed for the course evaluation and tested during the pandemic. By introducing and integrating the dashboard in a responsive panel, the functionalities of the applications were optimized, and at the same time, the evaluation process was facilitated and streamlined. After collecting the data through the interview guides, the authors were able to evaluate the level of influence on online education induced by the COVID-19 pandemic, and its influence on the courses throughout the studied period.

#### 4.2.2. Results of Monitoring and Evaluation of the Courses with EvalMathI Dashboard Software

Six online courses were monitored in the 2020/2021 academic year with the EvalMathI dashboard for this study. For each of the six monitored courses, the evaluation of the course was carried out by the analyzed parameters highlighting the percentage achieved by each course on various components. With the help of the dashboard in the EvalMathI system, the activity carried out for the six optional courses was monitored, following the evolution of several parameters, such as content, adaptability, skills, and involvement.

Q1. How did EvalMathI affect the evaluation process of the courses in an ERT situation?

**Figure 6:**
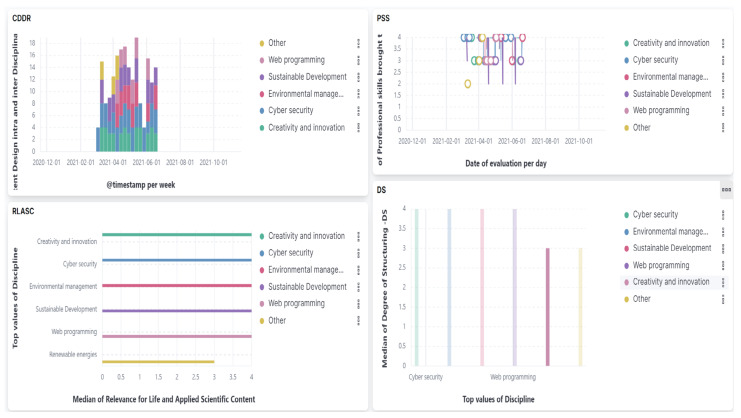
Final version of the responsive dashboard presenting Content Design Intra- and Interdisciplinary Relationship (CDDR), Relevance for Life and Applied Scientific Content (RLASC), Profession Student Skills (PSS), and Degree of Structuring (DS).

The final version of the responsive dashboard and EvalMathI, presented in Figure 6, represent two solutions, each of which performs different parts in the evaluation process. EvalMathI was developed in PHP and collects data from teachers and students using pages created for each type of user. The evaluations made by the experts are collected using Elasticsearch and then processed with Kibana. EvalMathI uses dynamic pages for creating dynamic content. The contents of the pages integrated with Elasticsearch are easy to analyze, being very intuitive and interactive. Field values can be easily seen with various filters. The responsive dashboard displays four panels, each of them answering questions regarding the evaluated courses—Content Design Intra- and Interdisciplinary Relationship (CDDR), Relevance for Life and Applied Scientific Content (RLASC), Profession Student Skills (PSS), and Degree of Structuring (DS).

Q2. What are the best EvalMathI dashboard tools regarding content evaluation? What does this response mean for the intra- and interdisciplinary relationship indicator (CDDR)?

**Figure 7:**
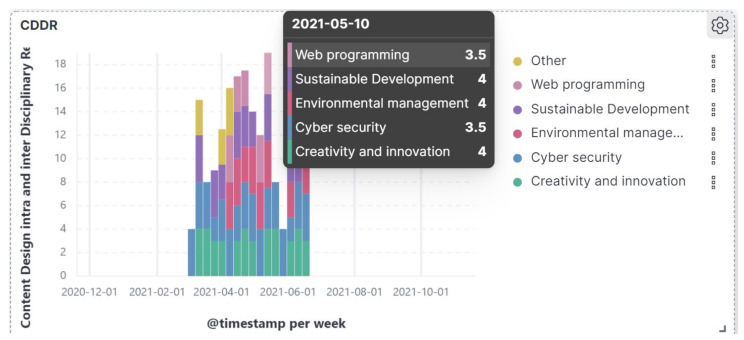
Responsive dashboard presenting Content Design Intra- and Interdisciplinary Relationship (CDDR).

In order to determine the content according to the CDDR indicator, the first panel, presented in Figure 7 from the responsive dashboard indicates the time interval of the courses’ development between March and June 2021. The results of the indicator have a median value between 3.5 and 4, which indicates that the course content sections at different times of evaluation corresponded by an 87.5% proportion.

Q3. How is the content of each course assessed through EvalMathI in terms of relevance and applied scientific content, coherence, and consistency?—What response does the responsive dashboard give regarding the relevance for life of each evaluated course, the RLASC indicator?

**Figure 8:**
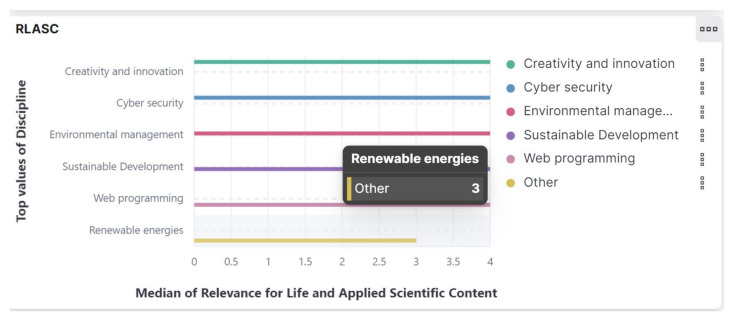
Responsive dashboard presenting Relevance for Life and Applied Scientific Content (RLASC).

The result presented in Figure 8 can be interpreted with the assumption that there were chapters or sections of this course for which the applied scientific content made up 75% or less of this indicator. The presence of the “Other” category for this discipline indicates a lack of completion of some fields. When fields are not completed, Elasticsearch interprets the results in this manner.

Q3. How is the content of each course assessed through EvalMathI in terms of relevance and applied scientific content, coherence, and consistency?—What response does the responsive dashboard give regarding the degree of structuring (DS)?

**Figure 9:**
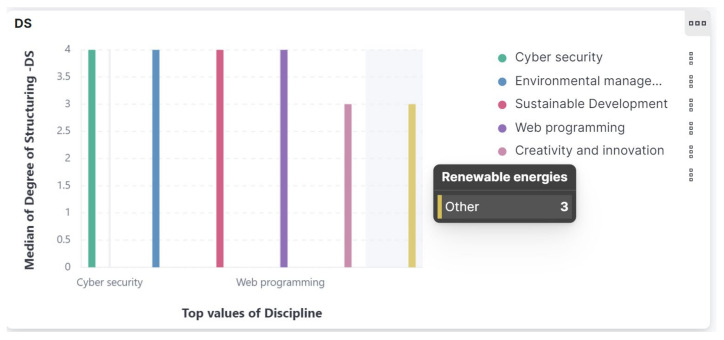
Responsive dashboard presenting Degree of Structuring (DS).

Regarding the Degree of Structuring (DS) and the Degree of Systematization (DSY), the indicators presented in Figure 9, the results extracted from Kibana are relative and investigations should be continued.

Q4. What skills were obtained by the students in completing the six courses? What response does the responsive dashboard give regarding the PSS indicator?

**Figure 10:**
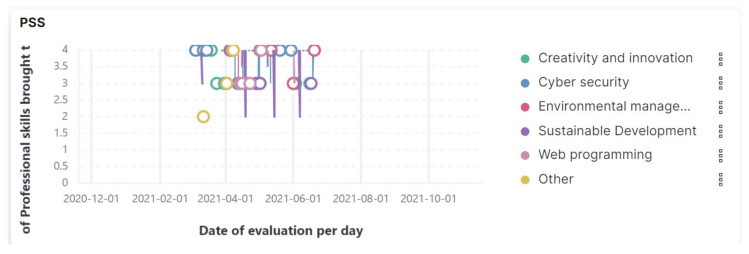
Responsive dashboard presenting Professional Student Skills (PSS).

Interactively, by positioning the cursor on each position, as it is presented in Figure 10, the authors found the responsive dashboard response for each course as the average value of the PSS indicator on the date it was completed. During the first stage, in the beta version, when data were collected with FAPA S1, the authors used Chart.js for representing the data in a responsive zone using responsive objects such as panels of courses, canvases, and so forth. In the second stage, the authors redefined the responsive dashboard concept because the application contained responsive charts. With Chart.js, the authors represented the responsive graphs and chose Chart.js because it set a certain value for that container. All the data were resized and the graph was responsive, responding at the width of the container, which was very useful in that stage. During the second stage, the authors added the Elastic Search component and the meaning of responsive was expanded.

## 5. Discussion

In the process of developing the LAEM model, the authors of the current study analyzed other learning models, such as the ADDIE model [43], the CIPP evaluation model [12], and the adaptive learning model [66]. Based on the presumption that the time factor is essential in ERT situations, in the present study, the authors proposed a model, called LAEM, which was adapted to the requirements of ERT for the academic level in which the evaluation block would give a direct answer to the teaching and learning activities.

The COVID-19 pandemic, perhaps one of the longest disruptive periods, except for times of wars, has irrevocably affected education and all related activities. Researchers around the world, whether they work in education or not, have studied the effects on all stakeholders involved in the education process. Many of them have also tried to look to the future and to estimate how the new face of education will look. A search in important databases such as the Web of Science Core Collection, Elsevier’s Scopus, The Directory of Open Access Journals, and Springer, and using the keywords “learning analytics” or “emergency remote teaching” or “responsive dashboard” led the authors to almost 600 results. A more refined search made within the open-access database journals published by MDPI, with the words “learning analytics” or “emergency remote teaching” or “responsive dashboard”, led the authors to 15 results, important papers published mainly in the Sustainability, Mathematics, Education Science, and Sensors journals during the COVID-19 period.

The result of the present study can be compared with 10 of these relevant papers [68,69,70,71,72,73,74,75,76,77]. While other researchers have analyzed the effects of ERT on high school teachers [68], state universities [69], and the challenges faced by educational institutions [70], for the proposed model, the developed EvalMathI system was tested to be able to answer questions Q1–Q4, questions that support the development of the model for the evaluation system (LAEM), and also validate the software instrument called EvalMathI. Other previous studies have shown that the teaching process in ERT can be improved mainly by improving the method of interactive communication and by optimizing the use of resources. Thus, for interactive communication between teachers and students, first, a new window was designed in EvalMathI, with which users can interact so that they can share information and communicate. The authors answered Q1—How useful is EvalMathI in evaluating courses in an ERT situation?—by introducing and integrating the dashboard in a responsive panel to facilitate and streamline the evaluation process. In addition, other researchers have previously analyzed students’ performance in an ERT situation [71], the challenges faced by math teachers in an ERT situation [72], the level of emotions in the learning process [75], the factors influencing home learning [78], and students’ emotions and the perception of teachers in ERT [76,77]. In this context, the present study analyzed the methodology of evaluation in ERT conditions and proposed a tool called EvalMathI, which was tested in a case study of six courses conducted in ERT at our university.

In another study [73], the authors dealt with the evaluation of the quality of courses in ERT situations, proposing a dashboard of indicators for decision making. However, the present study found results in the content of each course evaluated through EvalMathI in terms of the intra- and interdisciplinary relationship and the intra- and intercurricular relationship. In the present study, considering the relevance and the application of the content, EvalMathI highlighted two parameters for each discipline. Considering the degree of structuring and the degree of systematization of the content, the response to Q3 shows that EvalMathI evaluates each course. Thus, the authors could determine which courses had high scores, based on two indicators, the coherence and consistency of the contents. It was determined that the Cyber Security course obtained good scores, while the Web Programming course needs to be rethought to improve these indicators.

## 6. Conclusions

The sudden transition from face-to-face (F2F) education to online made in the education field in March 2020, without prior teacher training and without minimal knowledge of the tools used in online education by the students, has generated an educational flaw. Very few Romanian universities developed dedicated online platforms before the pandemic, which is why switching to online education actually meant switching to an ERT education system.

Based on their higher level of management, countries from western Europe have managed to overcome the pandemic waves, but at this time, Eastern Europe still faces major problems generated by the pandemic, problems that also affect education at all levels. Suddenly moving to an exclusively online system and using unprepared online teaching and evaluation modules at the academic level in the entire Romanian education system in the last year and a half has generated many unsolved problems.

According to the authors, future education will probably become more of a hybrid model in the academic field, while online secondary education will probably remain only an adjuvant for the teachers. This conclusion is based on the fact that online learning is second nature for students, and now, after a year and a half of online education, teachers are more prepared and trained. In addition, the transition to distance learning is now easier because different software companies have created resources that could help both teachers and students to migrate more easily to the online system. This conclusion is also supported by the results of another five papers published by the authors on the same subject during the 2020/2021 academic year [34,61,79,80,81], as well as by their expertise as members of the Romanian Agency for Quality Assurance in Higher Education (ARACIS) and of the Executive Agency for Higher Education, Research, Development and Innovation Funding (UEFISCDI), and as managers in the top management level of the university.

Another opinion of the authors of this study is that the future hybrid education system, composed of F2F and online education, will be more of an ERT system than a standard online education system based on eLearning platforms, which is why instruments, such as those presented in this paper, will become more necessary and useful in the future.

In the context of a future hybrid education system, the model and the application developed and proposed by the authors could solve some of the problems caused by this type of teaching, including online education in ERT conditions, because there is fear that by the end of 2021, the educational system will have to pass again through such an experience. This is one of the reasons the authors have tried to develop software tools for teachers, software that could solve some of the problems raised by teaching and evaluations exclusively online in ERT situations.

## Figures and Tables

**Table 1:** Summary of processed cases of variables. Reliability statistics.

Variable	Cronbach’s Alpha
Content Design Intra- and Interdisciplinary Relationship (CDDR)	0.964
Content Design Intra- and Intercurricular Relationship (CDCR)	0.829
Relevance for Life and Applied Scientific Content (RLASC)	0.835
Degree of Structuring (DS)	0.872
Degree of Systematization (DSY)	0.856
Coherence (CH)	0.834
Consistency (CS)	0.829

**Table 2:** The number of students who answered the questionnaire from the Mining and Science Faculties of the University of Petroșani.

Online Monitored Course	Academic Year	Number of Students/Course	% of total Number of Students/Academic Year
Sustainable Development	2019/2020	74	47%
Creativity and Innovation	2019/2020	41	26%
Environmental Management	2019/2020	18	11%
Renewable Energy	2019/2020	31	20%
Cyber Security	2019/2020	12	8%
Web Programming	2019/2020	14	9%
Sustainable Development	2020/2021	69	48%
Creativity and Innovation	2020/2021	49	34%
Environmental Management	2020/2021	58	41%
Renewable Energy	2020/2021	60	42%
Cyber Security	2020/2021	63	44%
Web Programming	2020/2021	40	28%
Total		529	

## Data Availability

The data presented in this study are available upon request from the first author.

## References

[1] Data.europa.eu. Education during COVID-19; Moving towards e-Learning. https://data.europa.eu/en/impact-studies/covid-19/education-during-covid-19-moving-towards-e-learning.

[2] Clark D. (2020). EU: Share of Individuals Doing an Online Course 2020. Published by D. Clark. https://www.statista.com/statistics/1099445/internet-use-in-schools-in-the-european-union/.

[3] Online Courses in Europe 2020|Statista Available Online. https://www.statista.com/.

[4] Verbert K., Govaerts S., Duval E., Santos J.L., Van Assche F., Parra G., Klerkx J. (2013). Learning dashboards: An overview and future research opportunities. Pers. Ubiquitous Comput..

[5] Duval E., Wiley D. (2010). Guest Editorial: Open Educational Resources. IEEE Trans. Learn. Technol..

[6] Chen G.D., Chang C.K., Wang C.Y. (2008). Ubiquitous learning website: Scaffold learners by mobile devices with information-aware techniques. Comput. Educ..

[7] Cen H., Koedinger K., Junker B. Automating Cognitive Model Improvement by A* Search and Logistic Regression. Proceedings of the AAAI 2005 Educational Data Mining Workshop, Carnegie Mellon University 5000 Forbes.

[8] Hodges C., Moore S., Lockee B., Trust T., Bond A. The Difference between Emergency Remote Teaching and Online Learning EDUCAUSE Review 2020. https://vtechworks.lib.vt.edu/handle/10919/104648.

[9] Information for Ohio State University Students, Faculty and Staff, the Ohio State University, Wexner Medical Center. https://safeandhealthy.osu.edu/.

[10] Coronavirus and Higher Education Resources Bryan Alexander Blog. https://bryanalexander.org/coronavirus/coronavirus-and-higher-education-resources.

[11] Zimmerman J. Coronavirus and the Great Online-Learning Experiment. The Chronicle of Higher Education, March 10. https://www.chronicle.com/article/Coronavirusthe-Great/248216.

[12] Stufflebeam D.L., Zhang G. (2017). Reporting Evaluation Findings in the Cipp Evaluation Model: How to Evaluate for Improvement and Accountability.

[13] Leitner P., Ebner M. Development of a Dashboard for Learning Analytics Development of a Dashboard for Learning Analytics in Higher Education. Proceedings of the Part II of Learning and Collaboration Technologies, Technology in Education: 4th International Conference, LCT 2017.

[14] Chen T., Peng L., Jing B., Wu C., Yang J., Cong G. (2020). The Impact of the COVID-19 Pandemic on User Experience with Online Education Platforms in China. Sustainability.

[15] Yu Y.-C., You S.-C.D., Tsai D.-R. Social Interaction Feedback System for the Smart Classroom. Proceedings of the 2012 IEEE International Conference on Consumer Electronics (ICCE).

[16] Pohl A., Bry F., Schwarz J., Gottstein M. Sensing the Classroom: Improving Awareness and Self-Awareness of Students with Backstage. Proceedings of the International Conference on Interactive and Collaborative Learning (ICL).

[17] Barr J., Gunawardena A. Classroom Salon: A Tool for Social Collaboration. Proceedings of the 43rd ACM Technical Symposium on Computer Science Education (Sigcse’12).

[18] Fagen W., Kamin S. (2012). Developing Device-independent Applications for Active and Collaborative Learning with the SLICE Framework. EdMedia+ Innovate Learning.

[19] Cuendet S., Bonnard Q., Kaplan F., Dillenbourg P. Paper interface design for classroom orchestration. Proceedings of the CHI’11 Extended Abstracts on Human Factors in Computing Systems (CHI EA’11).

[20] Son L.H. (2012). Supporting Reflection and Classroom Orchestration with Tangible Tabletops. Ph.D Thesis.

[21] Martinez Maldonado R., Kay J., Yacef K., Schwendimann B. An interactive teacher’s dashboard for monitoring groups in a multi-tabletop learning environment. Proceedings of the 11th International Conference on Intelligent Tutoring.

[22] Gutiérrez Rojas I., Crespo García R.M., Delgado Kloos C. (2011). Orchestration and feedback in lab sessions: Improvements in quick feedback provision. European Conference on Technology Enhanced Learning.

[23] Gutiérrez Rojas I., Crespo García R.M. Towards Efficient Provision of Feedback Supported by Learning Analytics. Proceedings of the 12th International Conference on Advanced Learning Technologies (ICALT).

[24] Morris M.R., Piper A.M., Cassanego T., Winograd T. Supporting Cooperative Language Learning: Issues in Interface Design for an Interactive Table, Stanford University Technical Report. https://hci.stanford.edu/cstr/reports/2005-08.pdf.

[25] Santos J.L., Govaerts S., Verbert K., Duval E. Goal-oriented visualizations of activity tracking: A case study with engineering students. Proceedings of the 2nd International Conference on Learning Analytics and Knowledge (LAK′12).

[26] Arnold K.E., Pistilli M.D. Course signals at Purdue: Using learning analytics to increase student success. Proceedings of the 2nd International Conference on Learning Analytics and Knowledge (LAK’12).

[27] Leony D., Crespo R.M., Perez-Sanagustin M., Parada G.H.A., de la Fuente Valentin L., Pardo A. Coverage Metrics for Learning- Event Datasets Based on Client-Side Monitoring. Proceedings of the IEEE 12th International Conference on Advanced Learning Technologies (ICALT).

[28] Dillenbourg P., Zufferey G., Alavi H., Jermann P., Do-Lenh S., Bonnard Q., Cuendet S., Kaplan F. (2011). Classroom Orchestration: The Third Circle of Usability.

[29] Bakker J., Holenderski L., Kocielnik R., Pechenizkiy M., Sidorova N.J. Stess@Work: From measuring stress to its understanding, prediction and handling with personalized coaching. Proceedings of the 2nd ACM Sighit International Health Informatics Symposium.

[30] Ferguson R. (2014). Learning Analytics: Fattori trainanti, sviluppi e storie. Ital. J. Educ. Technol..

[31] Suthers D. From contingencies to network-level phenomena: Multilevel analysis of activity and actors in heterogeneous networked learning environments. Proceedings of the Fifth International Conference on Learning Analytics and Knowledge.

[32] Nakahara J., Hisamatsu S., Yaegashi K., Yamauchi Y. Itree: Does the Mobile Phone Encourage Learners to Be More Involved in Collaborative Learning?. Proceedings of the 2005 Conference on Computer Support for Collaborative Learning.

[33] Kim I., Kim R., Kim H., Kim D., Han K., Lee P.H., Mark G., Lee U. (2019). Understanding smartphone usage in college classrooms: A long-term measurement study. Comput. Educ..

[34] Edelhauser E., Lupu-Dima L. (2020). Is Romania Prepared for eLearning during the COVID-19 Pandemic?. Sustainability.

[35] Kim S., Yun K., Park J., Choi J.Y. (2019). Skeleton-Based Action Recognition of People Handling Objects. Proceedings of the IEEE Winter Conference on Applications of Computer Vision (WACV).

[36] Pandea C., Witschela H.F., Montecchiaria A.M., Montecchiaria D. Hybrid Conversational AI for Intelligent Tutoring Systems. Proceedings of the AAAI Spring Symposium: Combining Machine Learning with Knowledge Engineering.

[37] Allen M., Maddison T., Kumaran M. (2017). Designing Online Asynchronous Information Literacy Instruction Using the ADDIE Model. Distributed Learning Chandos.

[38] Govaerts S., Verbert K., Duval E., Pardo A. The student activity meter for awareness and self-reflection. Proceedings of the Chi’12 ACM Annual Conference Extended Abstracts on Human Factors in Computing Systems Extended Abstracts.

[39] Dollár A., Steif P.S. Web-based Statics Course with Learning Dashboard for Instructors. Proceedings of the Computers and Advanced Technology in Education (CATE 2012).

[40] Ali L., Hatala M., Gašević D., Jovanović J. (2012). A qualitative evaluation of evolution of a learning analytics tool. Comput. Educ..

[41] Podgorelec V., Kuhar S. (2011). Taking Advantage of Education Data: Advanced Data Analysis and Reporting in Virtual Learning Environments. Electron. Electr. Eng..

[42] Leony D., Pardo A., de la Fuente Valentín L., Sánchez de Castro D., Delgado Kloos C. GLASS: A learning analytics visualization tool. Proceedings of the 2nd International Conference on Learning Analytics and Knowledge LAK’12.

[43] Scheuer O., Zinn C. How did the e-learning session go? The Student Inspector. Proceedings of the 13th International Conference on Artificial Intelligence and Education (AIED 2007).

[44] Lafford B.A. (2004). Review of Tell Me More Spanish. J. Lang. Learn. Technol..

[45] Kerly A., Ellis R., Bull S. (2007). CALMsystem: A Conversational Agent for Learner Modelling. Applications and Innovations in Intelligent Systems XV.

[46] Kobsa E., Dimitrova V., Boyle R. Using student and group models to support teachers in web-based distance education. Proceedings of the 10th International Conference on User Modeling.

[47] Santos J.L., Verbert K., Duval E. Empowering students to reflect on their activity with StepUp!: Two case studies with engineering students. Proceedings of the EFEPLE11 2nd Workshop on Awareness and Reflection in Technology-Enhanced Learning.

[48] Vieira C., Parsons P., Byrd V. (2018). Visual learning analytics of educational data: A systematic literature review and research agenda. Comput. Educ..

[49] Jivet I., Scheffel M., Specht M., Drachsler H. License to Evaluate: Preparing Learning Analytics Dashboards for Educational Practice. Proceedings of the 8th International Conference on Learning Analytics and Knowledge.

[50] Naranjo D.N., Prieto J.R., Moltó G., Calatrava A. (2019). A Visual Dashboard to Track Learning Analytics for Educational Cloud Computing. Sensors.

[51] Amazon AWS Cloudtrail. https://aws.amazon.com/cloudtrail/.

[52] Opsview Opsview Monitor. https://www.opsview.com/.

[53] Spectrum. https://spectrumapp.io/.

[54] SignalFx. https://signalfx.com/.

[55] SolarWinds AWS Cloud Monitoring. https://www.solarwinds.com/topics/aws-monitoring.

[56] Lonn S., Aguilar S.J., Teasley S.D. (2015). Investigating student motivation in the context of a learning analytics intervention during a summer bridge program. Comput. Hum. Behav..

[57] Sedrakyan G., Malmberg J., Verbert K., Järvelä S., Kirschner P.A. (2020). Linking learning behavior analytics and learning science concepts: Designing a learning analytics dashboard for feedback to support learning regulation. Comput. Hum. Behav..

[58] Irons A. (2007). Enhancing Learning through Formative Assessment and Feedback.

[59] (2020). Educația, o Șansă Pentru Valea Jiului!—POCU 122596. https://www.upet.ro/proiecte/122596/.

[60] (2021). Educația, o Șansă Pentru Valea Jiului!—POCU 122596. https://elearning-upet.ro/index.php#welcome.

[61] Edelhauser E., Lupu-Dima L. (2021). One Year of Online Education in COVID-19 Age, a Challenge for the Romanian Education System. Int. J. Environ. Res. Public Health.

[62] Verbert K., Duval E., Klerkx J., Govaerts S., Santos J.L. (2013). Learning Analytics Dashboard Applications. Am. Behav. Sci..

[63] Cho Y.S. Prospect for Learning Analytics to Achieve Adaptive Learning Model 2015, Seoul, Korea. https://www.slideshare.net/zzosang/prospect-for-learning-analytics-to-achieve-adaptive-learning-model.

[64] Community-led The Apache Way. https://www.apache.org/licenses/LICENSE-2.0.

[65] Pintrich P.R. (2004). A Conceptual Framework for Assessing Motivation and Self-Regulated Learning in College Students. Educ. Psychol. Rev..

[66] Corrin L., de Barba P. Exploring Students Interpretation of Feedback Delivered through Learning Analytics Dashboards. Proceedings of the Annual Conference of the Australian Society for Computers in Tertiary Education (Ascilite 2014), Rhetoric and Reality: Critical Perspectives on Educational Technology.

[67] Gormley C., Tong Z. Elasticsearch: The Definitive Guide.

[68] Gupta Y. Kibana Essentials.

[69] Seabra F., Teixeira A., Abelha M., Aires L. (2021). Emergency Remote Teaching and Learning in Portugal: Preschool to Secondary School Teachers’ Perceptions. Educ. Sci..

[70] Chierichetti M., Backer P. (2021). Exploring Faculty Perspectives during Emergency Remote Teaching in Engineering at a Large Public University. Educ. Sci..

[71] Colclasure B.C., Marlier A., Durham M.F., Brooks T.D., Kerr M. (2021). Identified Challenges from Faculty Teaching at Predominantly Undergraduate Institutions after Abrupt Transition to Emergency Remote Teaching during the COVID-19 Pandemic. Educ. Sci..

[72] Hidalgo G.I., Sánchez-Carracedo F., Romero-Portillo D. (2021). COVID-19 Emergency Remote Teaching Opinions and Academic Performance of Undergraduate Students: Analysis of 4 Students’ Profiles. A Case Study. Mathematics.

[73] Ní Fhloinn E., Fitzmaurice O. (2021). Challenges and Opportunities: Experiences of Mathematics Lecturers Engaged in Emergency Remote Teaching during the COVID-19 Pandemic. Mathematics.

[74] Mejía-Madrid G., Llorens-Largo F., Molina-Carmona R. (2020). Dashboard for Evaluating the Quality of Open Learning Courses. Sustainability.

[75] Nandi A., Xhafa F., Subirats L., Fort S. (2021). Real-Time Emotion Classification Using EEG Data Stream in E-Learning Contexts. Sensors.

[76] Ciordas-Hertel G.P., Rödling S., Schneider J., Di Mitri D., Weidlich J., Drachsler H. (2021). Mobile Sensing with Smart Wearables of the Physical Context of Distance Learning Students to Consider Its Effects on Learning. Sensors.

[77] Kohnke L., Zou D., Zhang R. (2021). Pre-Service Teachers’ Perceptions of Emotions and Self-Regulatory Learning in Emergency Remote Learning. Sustainability.

[78] Sosa Díaz M.J. (2021). Emergency Remote Education, Family Support and the Digital Divide in the Context of the COVID-19 Lockdown. Int. J. Environ. Res. Public Health.

[79] Edelhauser E., Lupu-Dima L. Managerial Research of Online Education in the Primary and Secondary Romanian Education System During COVID 19 Crisis. Proceedings of the MATEC Web of Conferences.

[80] Edelhauser E., Lupu-Dima L., Grigoras G. A Managerial Perspective over One Year of Online Education in Romania. A Case Study at the University of Petrosani. Proceedings of the MATEC Web of Conferences.

[81] Edelhauser E., Lupu-Dima L. A Comparative Study between Two Different Academic Years in the Online Education System for the Engineering and Economic Field. Proceedings of the 16th International Symposium in Management, Management, Innovation and Entrepreneurship in Challenging Global Times.

